# Supplementary material for: Simulation of undiagnosed patients with novel genetic conditions
Source: Nat Commun. 2023 Oct 12;14:6403. doi: 10.1038/s41467-023-41980-6 (PMC10570269; doi:10.1038/s41467-023-41980-6)
Supplement: Supplementary file 1 — Supplementary Information [file 41467_2023_41980_MOESM1_ESM.pdf]

Supplementary Information for

Simulation of undiagnosed patients with novel genetic conditions

Contents

Supplementary Notes

Supplementary Figures

S1 Phenotype terms with subtly differing prevalence between real and simulated patients S5
S2 Ability of computational approaches to rank causal genes differs across disease–gene categories . . . S6
S3 Shortest path distance between highly prioritzed genes and true causal gene by gene prioritization method . . . S7
S4 Overall ability of computational approaches to rank causal genes on entire simulated and real-world datasets . . . S8
S5 Pipeline components increase the difficulty of causal gene identification in simulated patients . . . S9
S6 Simulated patients have relatively fewer candidate genes from the Insufficiently Explanatory Gene Module . . . S10

Supplementary Notes

Supplementary Note 1: Undiagnosed Diseases Network Consortium Members. Maria T. Acosta, Margaret Adam, David R. Adams, Justin Alvey, Laura Amendola, Ashley Andrews, Euan A. Ashley, Mahshid S. Azamian, Carlos A. Bacino, Guney Bademci, Ashok Balasubramanyam, Dustin Baldridge, Jim Bale, Michael Bamshad, Deborah Barbouth, Pinar Bayrak-Toydemir, Anita Beck, Alan H. Beggs, Edward Behrens, Gill Bejerano, Hugo J. Bellen, Jimmy Bennet, Beverly Berg-Rood, Jonathan A. Bernstein, Gerard T. Berry, Anna Bican, Stephanie Bivona, Elizabeth Blue, John Bohnsack, Devon Bonner, Lorenzo Botto, Brenna Boyd, Lauren C. Briere, Elly Brokamp, Gabrielle Brown, Elizabeth A. Burke, Lindsay C. Burrage, Manish J. Butte, Peter Byers, William E. Byrd, John Carey, Olveen Carrasquillo, Thomas Cassini, Ta Chen Peter Chang, Sirisak Chanprasert, Hsiao-Tuan Chao, Gary D. Clark, Terra R. Coakley, Laurel A. Cobban, Joy D. Cogan, Matthew Coggins, F. Sessions Cole, Heather A. Colley, Cynthia M. Cooper, Heidi Cope, William J. Craigen, Andrew B. Crouse, Michael Cunningham, Precilla D'Souza, Hongzheng Dai, Surendra Dasari, Joie Davis, Jyoti G. Dayal, Matthew Deardorff, Esteban C. Dell'Angelica, Katrina Dipple, Daniel Doherty, Naghmeh Dorrani, Argenia L. Doss, Emilie D. Douine, Laura Duncan, Dawn Earl, David J. Eckstein, Lisa T. Emrick, Christine M. Eng, Cecilia Esteves, Marni Falk, Liliana Fernandez, Elizabeth L. Fieg, Paul G. Fisher, Brent L. Fogel, Irman Forghani, William A. Gahl, Ian Glass, Bernadette Gochuico, Rena A. Godfrey, Katie Golden-Grant, Madison P. Goldrich, Alana Grajewski, Irma Gutierrez, Don Hadley, Sihoun Hahn, Rizwan Hamid, Kelly Hassey, Nichole Hayes, Frances High, Anne Hing, Fuki M. Hisama, Ingrid A. Holm, Jason Hom, Martha Horike-Pyne, Alden Huang, Yong Huang, Wendy Introne, Rosario Isasi, Kosuke Izumi, Fariha Jamal, Gail P. Jarvik, Jeffrey Jarvik, Suman Jayadev, Orpa Jean-Marie, Vaidehi Jobanputra, Lefkothea Karaviti, Jennifer Kennedy, Shamika Ketkar, Dana Kiley, Gonench Kilich, Shilpa N. Kobren, Isaac S. Kohane, Jennefer N. Kohler, Deborah Krakow, Donna M. Krasnewich, Elijah Kravets, Susan Korrick, Mary Koziura, Seema R. Lalani, Byron Lam, Christina Lam, Grace L. LaMoure, Brendan C. Lanpher, Ian R. Lanza, Kimberly LeBlanc, Brendan H. Lee, Roy Levitt, Richard A. Lewis, Pengfei Liu, Xue Zhong Liu, Nicola Longo, Sandra K. Loo, Joseph Loscalzo, Richard L. Maas, Ellen F. Macnamara, Calum A. MacRae, Valerie V. Maduro, Rachel Mahoney, Bryan C. Mak, May Christine V. Malicdan, Laura A. Mamounas, Teri A. Manolio, Rong Mao, Kenneth Maravilla, Ronit Marom, Gabor Marth, Beth A. Martin, Martin G. Martin, Julian A. Martínez-Agosto, Shruti Marwaha, Jacob McCauley, Allyn McConkie-Rosell, Alexa T. McCray, Elisabeth McGee, Heather Mefford, J. Lawrence Merritt, Matthew Might, Ghayda Mirzaa, Eva Morava, Paolo M. Moretti, Mariko Nakano-Okuno, Stan F. Nelson, John H. Newman, Sarah K. Nicholas, Deborah Nickerson, Shirley Nieves-Rodriguez, Donna Novacic, Devin Oglesbee, James P. Orengo, Laura Pace, Stephen Pak, J. Carl Pallais, Christina GS. Palmer, Jeanette C. Papp, Neil H. Parker, John A. Phillips III, Jennifer E. Posey, Lorraine Potocki, Barbara N. Pusey, Aaron Quinlan, Wendy Raskind, Archana N. Raja, Deepak A. Rao, Anna Raper, Genecee Renteria, Chloe M. Reuter, Lynette Rives, Amy K. Robertson, Lance H. Rodan, Jill A. Rosenfeld, Natalie Rosenwasser, Francis

Rossignol, Maura Ruzhnikov, Ralph Sacco, Jacinda B. Sampson, Mario Saporta, Judy Schaechter, Timothy Schedl, Kelly Schoch, C. Ron Scott, Daryl A. Scott, Vandana Shashi, Jimann Shin, Edwin K. Silverman, Janet S. Sinsheimer, Kathy Sisco, Edward C. Smith, Kevin S. Smith, Emily Solem, Lilianna Solnica-Krezel, Ben Solomon, Rebecca C. Spillmann, Joan M. Stoler, Jennifer A. Sullivan, Kathleen Sullivan, Angela Sun, Shirley Sutton, David A. Sweetser, Virginia Sybert, Holly K. Tabor, Amelia L. M. Tan, Queenie K.-G. Tan, Mustafa Tekin, Fred Telischi, Willa Thorson, Cynthia J. Tifft, Camilo Toro, Alyssa A. Tran, Brianna M. Tucker, Tiina K. Urv, Adeline Vanderver, Matt Velinder, Dave Viskochil, Tiphanie P. Vogel, Colleen E. Wahl, Melissa Walker, Stephanie Wallace, Nicole M. Walley, Jennifer Wambach, Jijun Wan, Lee-kai Wang, Michael F. Wangler, Patricia A. Ward, Daniel Wegner, Monika Weisz-Hubshman, Mark Wener, Tara Wenger, Katherine Wesseling Perry, Monte Westerfield, Matthew T. Wheeler, Jordan Whitlock, Lynne A. Wolfe, Kim Worley, Changrui Xiao, Shinya Yamamoto, John Yang, Diane B. Zastrow, Zhe Zhang, Chunli Zhao, Stephan Zuchner

Supplementary Figures

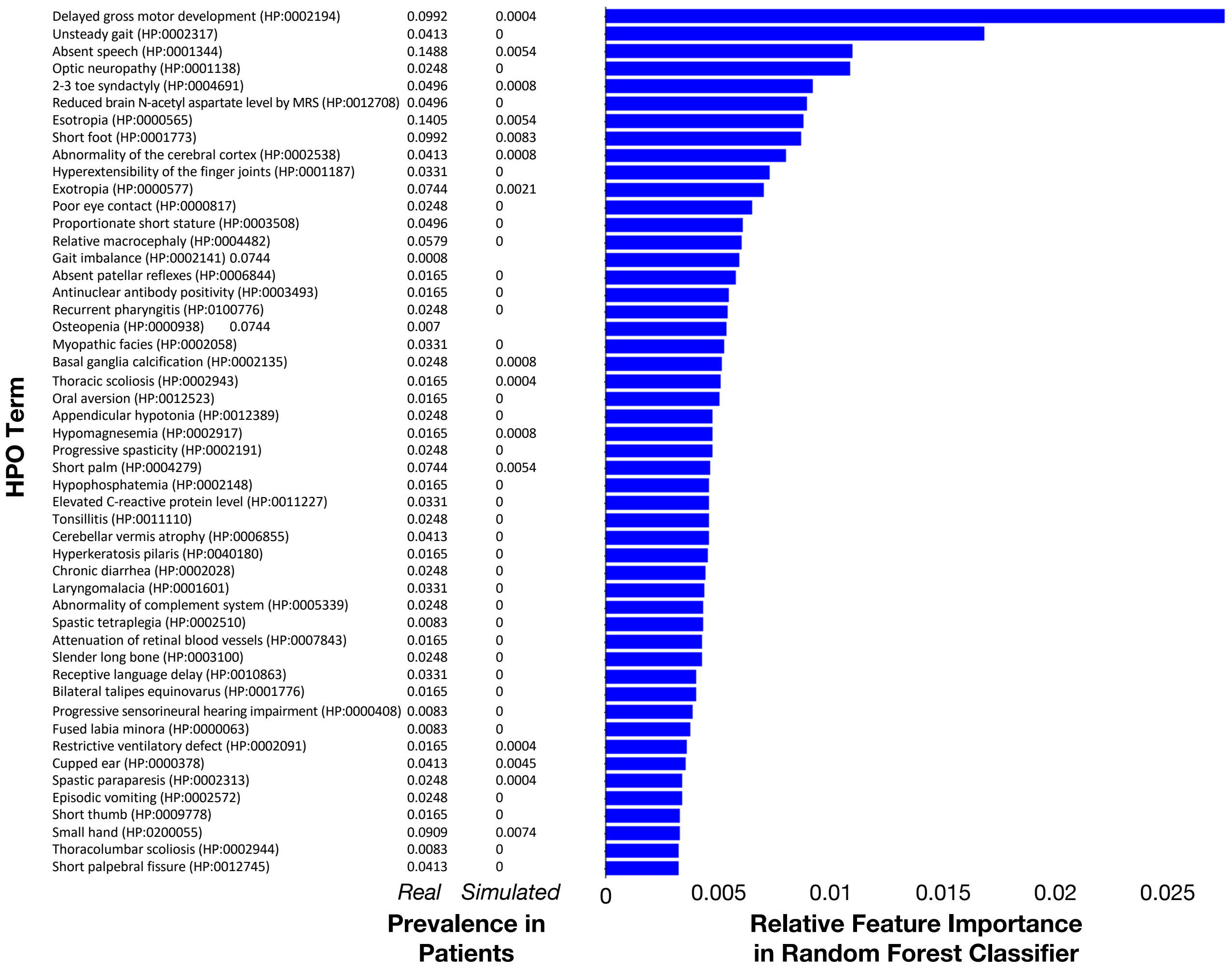

Figure S1: Phenotype terms with subtly differing prevalence between real and simulated patients. We implemented a random forest classifier to distinguish real from simulated patients in our matched cohort to identify which specific phenotype/genotype features were relatively unique to either group. Feature importance analysis of the resulting classifier demonstrates that there are specific codes that appear with prevalence of close to 0% in one of the groups even though they appear with low-prevalence (typically less than 5%) in the other group and therefore collectively provided great discriminatory power. The top such terms, their prevalence in both datasets, and relative feature importance are shown here. Source data are provided as a Source Data file.

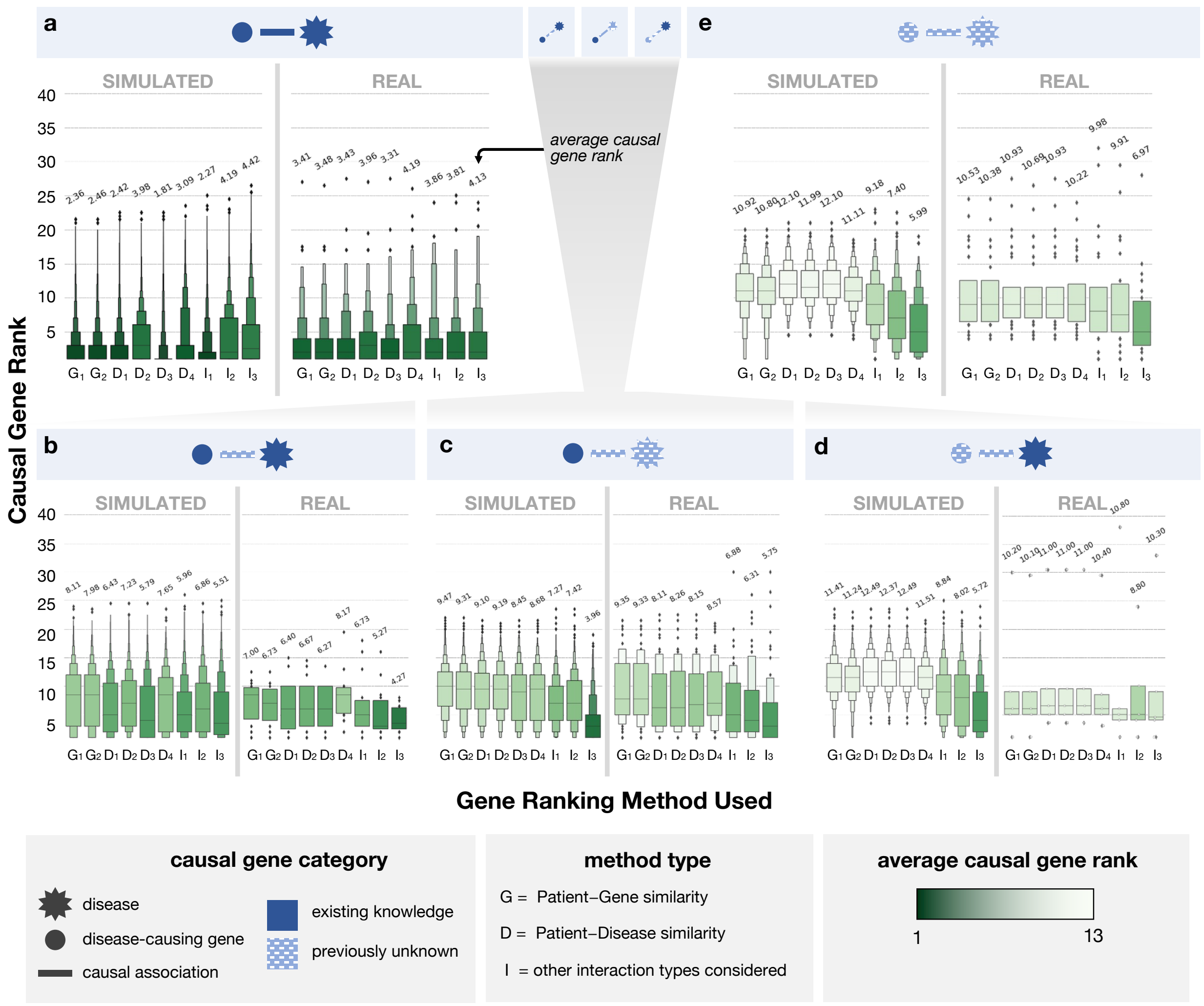

Figure S2: Ability of computational approaches to rank causal genes differs across disease–gene categories. We group simulated patients and real-world UDN patients into five categories based on their type of causal gene–disease association (patient counts in Table 1). These categories, described in detail in Figure 1b, are illustrated in the blue header bars above each plot and ordered decreasingly from left to right by the amount of existing knowledge of the association in the underlying knowledge graph. Each panel **a-e** shows performance on patients in a single category. We run nine gene ranking algorithms implemented in six prioritization tools on the phenotype terms and candidate gene list for each simulated and real-world patient within each causal gene–disease category. These algorithms are separated into those that directly consider patient–gene phenotypic similarity (G_1: Phrank–Gene, G_2: ERIC–Gene), those that compute patient–disease phenotypic similarity (D_1: Phrank–Disease, D_2: ERIC–Disease, D_3: Phenomizer, D_4: LIRICAL), and those that consider additional interaction edges, such as gene–gene edges, interactions in other species, or predicted edges (I_1: Phenolyzer, I_2: HiPhive, I_3: ERIC-Predicted). We show here boxen plots depicting the distribution of the ranks of each patient's causal genes. The median is shown on each boxen by a horizontal line segment, and the innermost box is drawn at the first and third quartiles, as in the conventional boxplot. The boxes are nested, with each successive box corresponding to a smaller quantile interval. The average rank of the causal gene is italicized above each boxen. Each boxen is also colored according to the average causal gene rank. Source data are provided as a Source Data file.

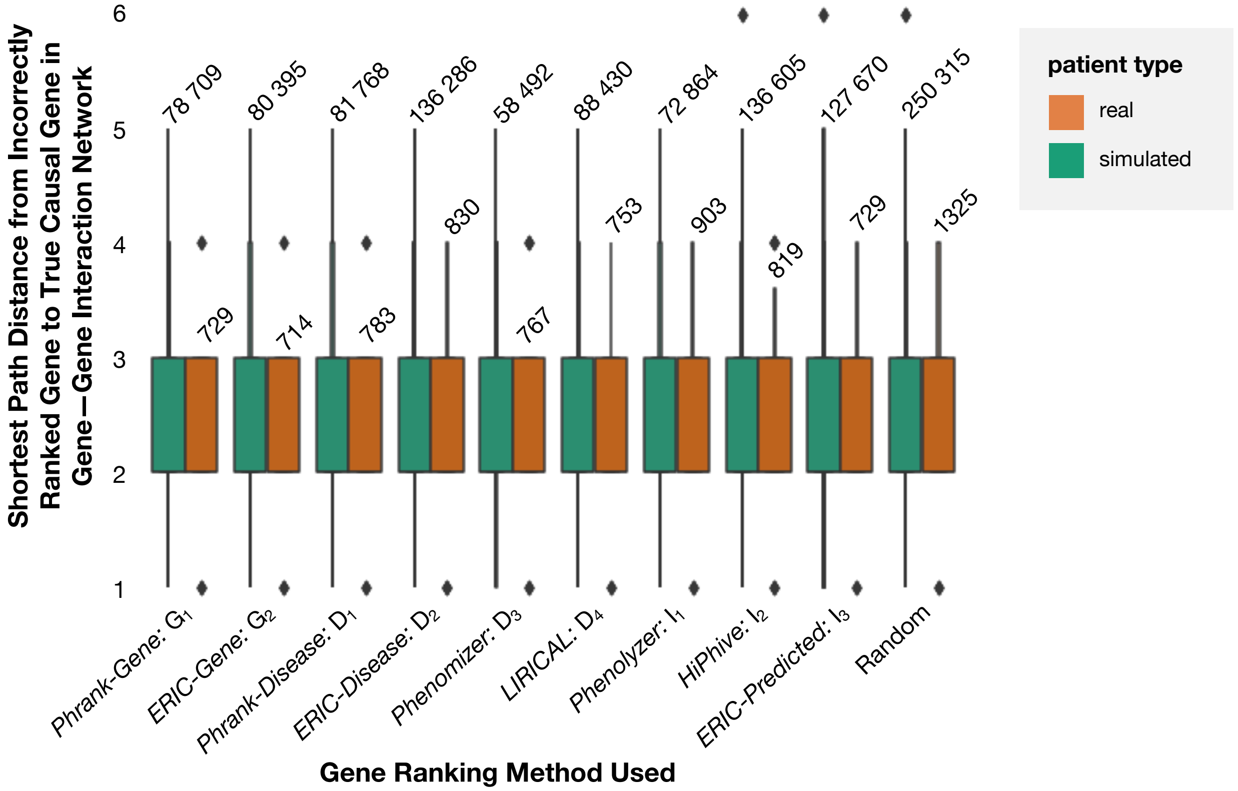

Figure S3: Shortest Path Distance between True Causal Gene and Incorrectly Prioritized Genes by Gene Prioritization Method. We show boxen plots depicting the distance (number of edges on shortest path) between the true causal gene and all genes incorrectly prioritized above the true causal gene per patient in a protein–protein and transcription factor interaction network curated from the Human Protein Reference Database and the Human Transcriptional Regulation Interactions Database. Real and simulated patients where the causal gene was ranked first or where no gene–gene path existed between the top-ranked and causal gene are excluded per gene prioritization method. The shortest path distances between the causal gene and the incorrectly prioritized genes are similar across methods, including those that do not consider gene–gene interaction edges, and are largely indistinguishable within each prioritization method. The innermost box of each boxen plot is drawn at the first and third quartiles, as in the conventional boxplot. The median is a horizontal line, which coincides with the first quartile in all depicted boxens. The boxes are nested, with each successive box corresponding to a smaller quantile interval. The numbers above each boxen denote the number of incorrectly prioritized genes evaluated for each method and patient type. Source data are provided as a Source Data file.

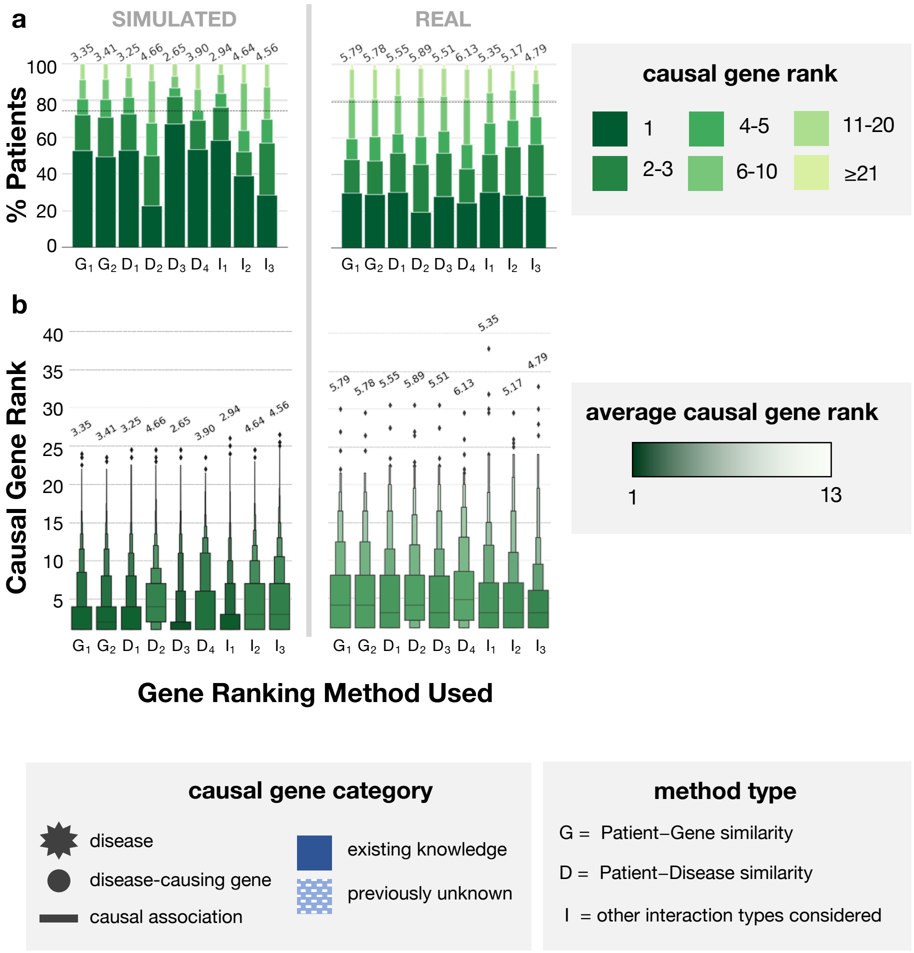

Figure S4: Overall ability of computational approaches to rank causal genes on entire simulated and real-world datasets. We run the gene ranking methods on the phenotype terms and candidate genes for all simulated patients (n = 42,680) and real-world patients (n = 248) in the cohorts, and we show here the ability of these methods to correctly rank each patient's causal gene by showing the proportion of patients with the causal gene ranked within the top k genes (**a**) and the distribution of overall causal gene ranks as boxen plots (**b**). The average rank of the causal gene is italicized above each bar. The median is shown on each boxen by a horizontal line segment, and the innermost box is drawn at the first and third quartiles. The boxes are nested, with each successive box corresponding to a smaller quantile interval. Notably, the performance of the algorithms on the entire cohort does not reflect heterogeneity in performance across disease–gene categories as shown in Figure 4. Specifically, the gene prioritization algorithms have better relative performance on simulated patients versus real-world patients in the one category where the disease is known and caused by a known associated causal gene. The overall prevalence of patients in this category is much higher than the prevalence of patients in all other novelty categories (Table 1). This likely contributes to the seemingly higher performance of the gene prioritization algorithms on the entire simulated patient cohort, which is not reflected in the stratified performance across disease–gene categories. Source data are provided as a Source Data file.

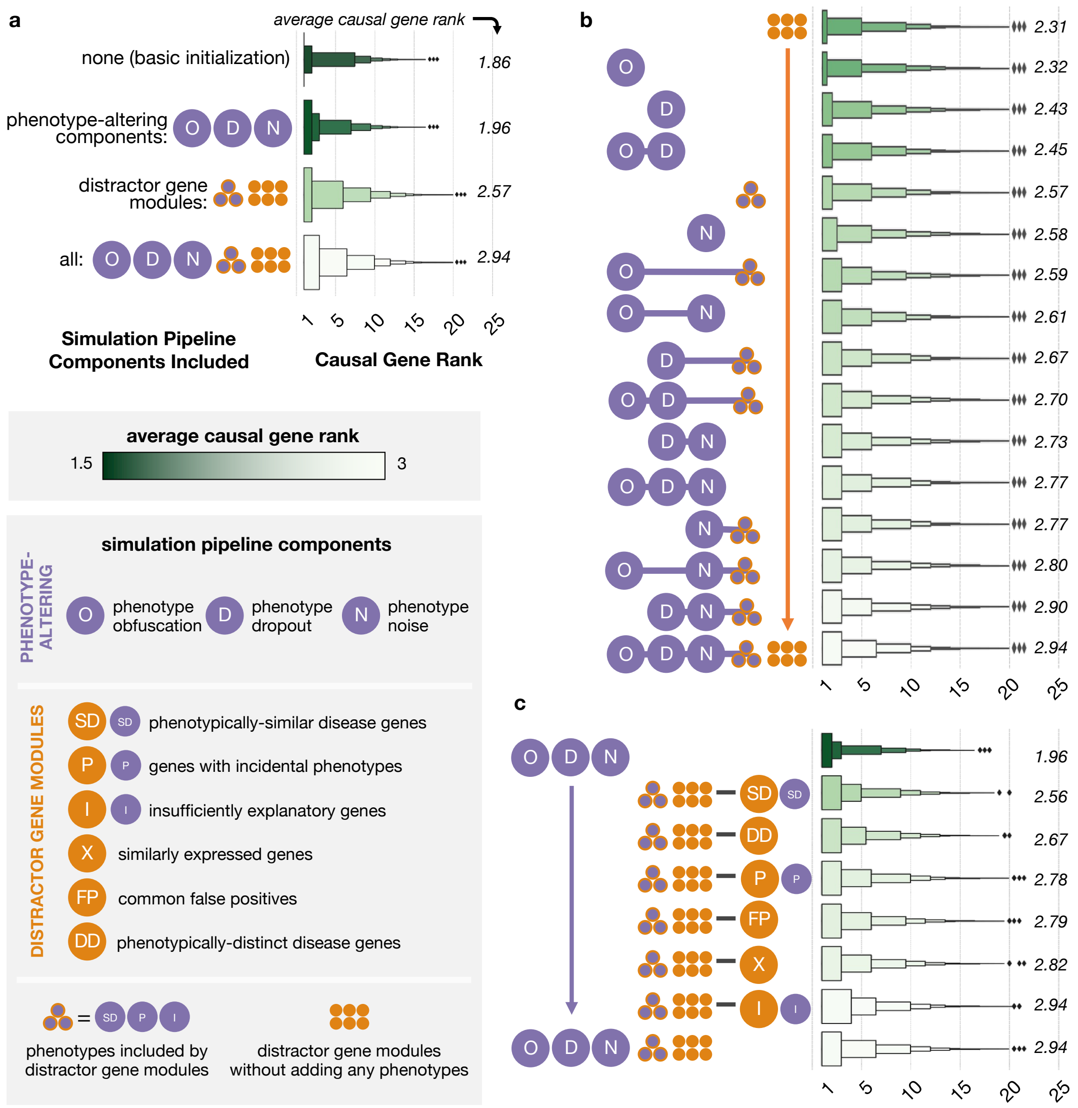

Figure S5: Pipeline components increase the difficulty of causal gene identification in simulated patients. We run a gene prioritization algorithm on patients simulated by our pipeline when varying subsets of pipeline components are included. We show boxen plots visualizing the distribution of the ranks of each patient's causal genes (horizontal axis for all plots) when different components of the simulation pipeline are included (vertical axis for all plots). The average rank of the causal gene is listed in italics next to each boxen plot. We show gene prioritization performance on simulated patients produced when the following components are included in the simulation pipeline: **a.** no phenotype- nor gene-based components (i.e., candidate genes sampled randomly and phenotype terms unaltered from initialization), all standalone phenotype-altering components alone, all distractor gene modules alone, or all pipeline components together; **b.** a "gene-only" version of distractor gene modules and each possible combination of subsets of phenotype-altering components; **c.** all three standalone phenotype-altering components and all but one distractor gene module at a time. Note that in b, horizontal purple lines in the vertical axis labels are for visual clarity, whereas in c, horizontal black lines in the vertical axis signify set difference. Source data are provided as a Source Data file.

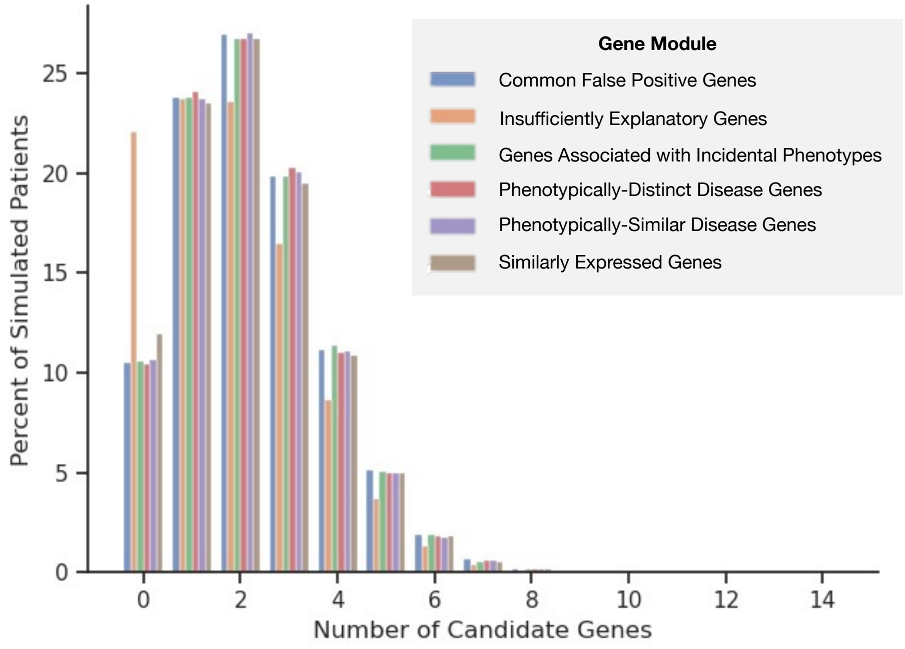

Figure S6: Simulated patients have relatively fewer candidate genes from the Insufficiently Explanatory Gene Module. In the gene module ablation experiment, we run the simulation pipeline with an equal probability of sampling each distractor gene module and perform an ablation of each of the six distractor gene modules by removing a single module at a time. Despite sampling each module with equal probability, there are fewer patients with candidate genes added by the Insufficiently Explanatory Gene Module compared to other gene modules in our initial simulated patient cohort. Insufficiently explanatory genes may not be added to a patient if there are no qualifying non-disease genes that are associated with a strict subset of low prevalence phenotypes from the simulated patient's true disease (See Methods). The lower prevalence of Insufficiently Explanatory genes may explain why removal of these genes does not change gene prioritization performance as shown in Figure 5c. Source data are provided as a Source Data file.
