# Supplementary material for: Simulation of undiagnosed patients with novel genetic conditions
Source: Nat Commun. 2023 Oct 12;14:6403. doi: 10.1038/s41467-023-41980-6 (PMC10570269; doi:10.1038/s41467-023-41980-6)
Supplement: Supplementary file 2 — Reporting Summary [file 41467_2023_41980_MOESM2_ESM.pdf]

Corresponding author(s): Shilpa Kobren, Isaac Kohane

Last updated by author(s): Jul 28, 2023

Reporting Summary

Nature Portfolio wishes to improve the reproducibility of the work that we publish. This form provides structure for consistency and transparency in reporting. For further information on Nature Portfolio policies, see our Editorial Policies and the Editorial Policy Checklist.

Statistics

For all statistical analyses, confirm that the following items are present in the figure legend, table legend, main text, or Methods section.

n/a	Confirmed	
☐	☒	The exact sample size (*n*) for each experimental group/condition, given as a discrete number and unit of measurement
☒	☐	A statement on whether measurements were taken from distinct samples or whether the same sample was measured repeatedly
☐	☒	The statistical test(s) used AND whether they are one- or two-sided<br>*Only common tests should be described solely by name; describe more complex techniques in the Methods section.*
☒	☐	A description of all covariates tested
☐	☒	A description of any assumptions or corrections, such as tests of normality and adjustment for multiple comparisons
☐	☒	A full description of the statistical parameters including central tendency (e.g. means) or other basic estimates (e.g. regression coefficient) AND variation (e.g. standard deviation) or associated estimates of uncertainty (e.g. confidence intervals)
☐	☒	For null hypothesis testing, the test statistic (e.g. *F*, *t*, *r*) with confidence intervals, effect sizes, degrees of freedom and *P* value noted<br>*Give P values as exact values whenever suitable.*
☒	☐	For Bayesian analysis, information on the choice of priors and Markov chain Monte Carlo settings
☒	☐	For hierarchical and complex designs, identification of the appropriate level for tests and full reporting of outcomes
☒	☐	Estimates of effect sizes (e.g. Cohen's *d*, Pearson's *r*), indicating how they were calculated

Our web collection on statistics for biologists contains articles on many of the points above.

Software and code

Policy information about availability of computer code

Data collection	The published paper "Commonalities across computational workflows for uncovering explanatory variants in undiagnosed cases" describes the computational workflow used to generate sets of phenotypes and candidate genes for patients in the Undiagnosed Diseases Network. All computational software used in the creation of the simulation dataset is described in our open source github repository: https://github.com/EmilyAlsentzer/rare-disease-simulation.
Data analysis	We leverage Python 3.6.7, SciPy 1.5.4, and Obonet 0.3.0 for our analysis. We provide a full list of all software and version numbers in the requirements.txt file in our open source github repository: https://github.com/EmilyAlsentzer/rare-disease-simulation.

For manuscripts utilizing custom algorithms or software that are central to the research but not yet described in published literature, software must be made available to editors and reviewers. We strongly encourage code deposition in a community repository (e.g. GitHub). See the Nature Portfolio guidelines for submitting code & software for further information.

Data

Policy information about availability of data

All manuscripts must include a data availability statement. This statement should provide the following information, where applicable:

- Accession codes, unique identifiers, or web links for publicly available datasets
- A description of any restrictions on data availability
- For clinical datasets or third party data, please ensure that the statement adheres to our policy

The simulated patient dataset and all intermediate data used in its creation have been deposited in Harvard Dataverse under accession code https://doi.org/10.7910/DVN/ANFOR3. Anonymized UDN data has been deposited in dbGaP (accession phs001232) and PhenomeCentral. Phenotypes and causal variants and genes related to UDN diagnoses are also shared publicly in ClinVar: www.ncbi.nlm.nih.gov/clinvar/submitters/505999/. Our simulation process and analyses leverage the following external databases: Human Phenotype Ontology (https://hpo.jax.org/app/), HPO Annotations (github.com/drseb/HPO-archive/tree/master/hpo.ann522
otations.monthly/2015-01-28 14-15-03/archive/annotation and github.com/drseb/HPO-arc523
hive/tree/master/2014-2015/2015 week 4/annotations/artefacts), Orphanet (orphadata.org), Unified Medical Language System (nlm.nih.gov/research/umls/index.html), Human Transcriptional Regulation Interactions Database (available in the Phenolyzer Github at https://github.com/WGLab/phenolyzer), Human Protein Reference Database (https://www.hprd.org/; also available in the Phenolyzer Github), CADD (https://cadd.gs.washington.edu/), and Ensembl BioMart (https://useast.ensembl.org/info/data/biomart/index.html). All datasets used in our analyses can be found on the Harvard Dataverse. Furthermore, the data needed to recreate the figures can be found in the "Source Data" section of our Harvard Dataverse.

Human research participants

Policy information about studies involving human research participants and Sex and Gender in Research.

Reporting on sex and gender	Our work did not consider sex and/or gender in the study design because we do not separately consider nor restrict to rare diseases that affect primarily males or primarily females during the simulation process. However, we do note that there are 4,033 simulated patients and 18 UDN patients with causal genes on an X chromosome of 42,680 and 248 total patients respectively. Recessive manifestations of these diseases would be less prevalent in females.
Population characteristics	Approximately 81% of patients in the UDN cohort listed their race as White, 5% listed their race as Black or African American, 9% listed their race as Asian, and the remaining 5% listed their race as Other. 19% are Hispanic or Latino, 71% are not Hispanic or Latino, and the remaining 10% have unknown ethnicity. The patients vary in age from a few months old to over 65 years old with 75.8% of patients under the age of 5. The presenting symptoms for UDN patients vary considerably, but the top 5 classes of primary presenting symptoms for patients in the UDN cohort are: neurologic (53%), musculoskeletal (15%), Other (13%), cardiac (3%), and immunologic (3%).
Recruitment	In this study, we do not actively enlist patients. Instead, we utilize patient information from the Undiagnosed Diseases Network (UDN). Potential participants (or their legal guardians) can discover the UDN through various means, such as the UDN website, promotional materials, or another participant's referral. Health care professionals unaffiliated with the UDN might become aware of it via the UDN website, publicity, their peers, or medical conferences and publications. Medical practitioners at UDN-affiliated Clinical Sites can recommend their patients for assessment. Individuals (or their legal guardians) sign up and submit applications to the UDN via the Gateway, which the Coordinating Center manages. Consequently, the patient demographic within the UDN is more likely to reflect those who have access to these recruitment methods and/or reside in regions with a UDN clinical site.
Ethics oversight	The Undiagnosed Diseases Network study is approved by the National Institutes of Health institutional review board (IRB), which serves as the central IRB for the study (Protocol 15HG0130).

Note that full information on the approval of the study protocol must also be provided in the manuscript.

Field-specific reporting

Please select the one below that is the best fit for your research. If you are not sure, read the appropriate sections before making your selection.

☒ Life sciences ☐ Behavioural & social sciences ☐ Ecological, evolutionary & environmental sciences

For a reference copy of the document with all sections, see nature.com/documents/nr-reporting-summary-flat.pdf

Life sciences study design

All studies must disclose on these points even when the disclosure is negative.

Sample size	The sample size for the Undiagnosed Diseases Network patients was dictated by the number of diagnosed rare disease patients in the UDN. We evaluated all diagnosed patients with phenotypes and at least 5 candidate genes at the time of the study. For our simulations , we generated 20 patients for each of the available monogenic diseases in orphanet that met the criteria described in our Methods section. This

	resulted in a set of 42,680 simulated patients, which we leveraged in our downstream analyses. However, we note that the number of patients simulated per disease is a parameter, and users of our simulation pipeline can adjust this number to suit their needs.
Data exclusions	We exclude Undiagnosed Diseases Network patients who do not have any phenotypes, do not have at least 5 candidate genes, and who are not diagnosed. This filtering is necessary to generate a real-world UDN cohort that can be used to validate gene prioritization algorithms.
Replication	Our simulation pipeline is publicly available at https://github.com/EmilyAlsentzer/rare-disease-simulation, which enables the community to reproduce our findings.
Randomization	Randomization is not necessary for this study as there is no treatment/control groups nor does the study attempt to assess causality.
Blinding	Blinding is not necessary for this study as there is no assignment to treatment/control groups.

Reporting for specific materials, systems and methods

We require information from authors about some types of materials, experimental systems and methods used in many studies. Here, indicate whether each material, system or method listed is relevant to your study. If you are not sure if a list item applies to your research, read the appropriate section before selecting a response.

Materials & experimental systems

n/a	Involved in the study
☒	☐ Antibodies
☒	☐ Eukaryotic cell lines
☒	☐ Palaeontology and archaeology
☒	☐ Animals and other organisms
☐	☒ Clinical data
☒	☐ Dual use research of concern

Methods

n/a	Involved in the study
☒	☐ ChIP-seq
☒	☐ Flow cytometry
☒	☐ MRI-based neuroimaging

Clinical data

Policy information about clinical studies

All manuscripts should comply with the ICMJE guidelines for publication of clinical research and a completed CONSORT checklist must be included with all submissions.

Clinical trial registration	The Undiagnosed Diseases Network (UDN) study registration can be found at https://clinicaltrials.gov/ct2/show/NCT02450851. Note that while our paper leverages data from the UDN study, we are not the first to report the findings from the UDN study and therefore are omitting the CONSORT checklist.
Study protocol	The UDN manual of operations can be found at https://undiagnosed.hms.harvard.edu/research/udn-manual-of-operations/
Data collection	The Undiagnosed Diseases Network began recruitment in 2015. Recruitment is continuous. Once a patient is accepted into the UDN, he/she travels to a UDN clinical site for a 5-day clinical and research visit.
Outcomes	The UDN has the following outcome measures: (1) Improve the level of diagnosis and care for patients with undiagnosed diseases through the development of common and site-specific protocols designed by an enlarged community of investigators, (2) Facilitate research into the etiology of undiagnosed diseases, by collecting and sharing standardized, high-quality clinical and laboratory data including genotyping, phenotyping, and documentation of environmental exposures, and (3) Create an integrated and collaborative research community across multiple clinical sites and between laboratory and clinical investigators prepared to investigate the pathophysiology of these new and rare diseases, the impact of the diagnostic process on patients and families, and share this understanding to identify improved options for optimal patient management.
